# Hematopoietic organoids: Opportunities and challenges in modeling human hematopoiesis and diseases *in vitro*

**DOI:** 10.1016/j.stemcr.2025.102755

**Published:** 2026-01-02

**Authors:** Liming Du, Yuxin Huang, Feng Liu

**Affiliations:** 1 Shandong Provincial Key Laboratory of Development and Regeneration, School of Life Sciences, Qilu Hospital (Qingdao), Cheeloo College of Medicine, Shandong University, Qingdao, China; 2 Key Laboratory of Organ Regeneration and Reconstruction, State Key Laboratory of Membrane Biology, Institute for Stem Cell and Regeneration, Institute of Zoology, University of Chinese Academy of Sciences, Chinese Academy of Sciences, Beijing, China

**Keywords:** hematopoietic organoid, hematopoiesis, hematopoietic stem cells, organoid

## Abstract

Previous studies on hematopoiesis were mainly conducted in model animals. However, direct investigation of human hematopoiesis remains challenging due to limited access to human samples and ethical concerns. Traditional two-dimensional culture systems have provided valuable opportunities to study human hematopoiesis, but they fail to fully recapitulate the behaviors of hematopoietic cells and their interactions with niche cells as observed *in vivo*. In recent years, organoid technologies have emerged as a powerful approach for modeling hematopoietic development, maintenance, and diseases. By mimicking the key architectural and functional characteristics of native hematopoietic tissues, hematopoietic organoids (HOs) offer promising platforms for studying developmental hematopoiesis, modeling hematological diseases, performing drug screening, and generating functional hematopoietic cells. In this review, we summarize recent progress in HO construction, explore their potential applications in both basic research and clinical translation, and discuss current opportunities and remaining challenges in generating physiologically relevant HO models.

## Teaser

Hematopoietic organoids are emerging as powerful 3D models that overcome the limitations of traditional 2D cultures. In this review, Feng Liu and colleagues explore how these organoids are revolutionizing the study of human blood development, disease, and drug discovery.

## Introduction

Hematopoietic tissues are responsible for the development, maintenance, and regeneration of hematopoietic system. Among the various hematopoietic cell types existing in these tissues, hematopoietic stem cells (HSCs) are central to the whole process of hematopoiesis, and the study of HSC biology is fundamental to understanding both the development and maintenance of the hematopoietic systems. Since their initial identification in mice in the 1950s (Jacobson et al., 1950), much of our current knowledge of HSC biology and hematopoiesis has been derived from animal models or two-dimensional (2D) *in vitro* culture systems (Comazzetto et al., 2019; Ding et al., 2012; Gao et al., 2022; Khan et al., 2016; Li et al., 2015; Mendez-Ferrer et al., 2010; Orkin and Zon, 2008; Silberstein et al., 2016; Solaimani Kartalaei et al., 2015; Wang et al., 2013; Wei and Frenette, 2018; Winkler et al., 2010; Xu et al., 2018; Xue et al., 2017; Zhang et al., 2003, 2015a, 2017, 2019, 2022; Zheng et al., 2022; Zovein et al., 2008). During mammalian embryogenesis, HSCs originate from hemogenic endothelium (HE), a specialized type of endothelial cell in the dorsal aorta of aorta-gonad-mesonephros (AGM) region, through a process termed endothelial-to-hematopoietic transition (EHT) (Zhang et al., 2018b). After their migration to the fetal liver, where HSCs undergo maturation, expansion, and differentiation (Gao et al., 2022), they finally colonize in the bone marrow to sustain the lifelong hematopoiesis through their self-renewal and multilineage differentiation abilities (Morrison et al., 1995; Orkin and Zon, 2008). Under stress conditions or following bone marrow transplantation, HSCs are rapidly activated and drive hematopoietic regeneration (Bonnefoix and Callanan, 2010; Calvanese et al., 2019; Kiel et al., 2005; Lv et al., 2021; Morrison and Scadden, 2014; Zhou et al., 2017a), highlighting their clinical importance in treating multiple hematological disorders (Armitage, 1994; Gratwohl et al., 2015; Lee, 2024; Niederwieser et al., 2022). Notably, the architectural and functional characteristics of specific hematopoietic tissues ensure the proper development and maintenance of HSCs within their distinct niche.

Besides HSCs, differentiated hematopoietic cells, such as T cells and macrophages, are generated in specific hematopoietic tissues and play critical roles in the pathogenesis of immune disorders and cancers, suggesting the potential application of hematopoietic cell-based strategies in establishing personalized therapies for various immune-related diseases (Labanieh and Mackall, 2023; Weber et al., 2020). Conversely, dysregulation of hematopoiesis can lead to the occurrence of hematological disorders and malignancies, further emphasizing the importance of understanding human hematopoiesis in maintaining hematopoietic health and developing targeted interventions.

Animal models and traditional 2D culture systems using human-derived primary cells have provided essential tools to study hematopoiesis. However, due to species-specific differences and the limited physiological complexity of these models, many findings are not directly translatable to human biology. Particularly, conventional 2D culture systems are unable to replicate the key spatial and mechanical features of *in vivo* hematopoietic tissues (Khan et al., 2023), including three-dimensional (3D) structures, cellular diversity, viscoelastic properties, vascularization, and others. Thus, there remains a critical need for advanced models that more accurately recapitulate the human hematopoietic tissues. The development of hematopoietic organoid (HO) systems has addressed many of these limitations. HOs are 3D multicellular aggregates that recapitulate organotypic structures and functions of hematopoietic tissues (Frenz-Wiessner et al., 2024; Ng et al., 2016, 2024; Olijnik et al., 2024), by which scientists can explore the underlying mechanisms of human hematopoiesis, screen drugs for treatment of hematopoietic diseases, and generate transplantable HSCs or immune cells *in vitro* for clinical applications.

In this review, we first introduce traditional 2D culture models used for hematopoietic investigations. We then focus on recent studies on 3D HO technologies, highlighting their potential applications in both basic research and clinical trials. Finally, we discuss the current challenges and limitations that remain in the field and propose potential strategies for further improving HO models.

## Traditional cell culture-based models to study HSCs

### Transitioning from 2D to 3D cell culture

*In vitro* cell culture was established in the mid-twentieth century. Since then, numerous cell types have been successfully cultured in 2D plastic petri dishes, including HSCs (Wilkinson et al., 2019, 2020), immune cells (Bar-Ephraim et al., 2020; Look et al., 2023; Schmitt and Zuniga-Pflucker, 2002), mesenchymal stem/stromal cells (MSCs) (Du et al., 2019; Ren et al., 2009), endothelial cells (Nguyen et al., 2013; Shamir and Ewald, 2014; Zheng et al., 2012), cancer cells (Deng et al., 2022; Drost and Clevers, 2018), and others. These 2D culture systems serve as valuable tools for visualizing, manipulating, and analyzing cell behavior at both cellular and molecular levels.

However, *in vivo*, primary cells reside within complex 3D microenvironments where they keep continuous contact with a variety of neighbor cells and extracellular matrix (ECM). The native niches are characterized by a well-defined ECM composition (Fischer et al., 2023; Saraswathibhatla et al., 2023; Zhu et al., 2019), oxygen and nutrient gradients (Luo et al., 2022), 3D spatial organization, and distinct tissue stiffness and viscoelasticity (De Belly et al., 2022; Saraswathibhatla et al., 2023). Furthermore, the location of cells within tissues is highly organized and follows specific patterns (Bar-Ephraim et al., 2020; Corro et al., 2020; Drost and Clevers, 2018; Griffith and Wagner, 2017). The absence of these physiological architectures in 2D cultures often alters cell polarity, morphology, gene expression, and function, ultimately limiting the biological relevance and translational applicability of these models (Hofer and Lutolf, 2021; Khan et al., 2023).

Therefore, efforts to improve 2D culture systems have led to several innovations. A key milestone was reached in 1975 with the development of the first co-culture system, which enabled primary human keratocytes to form a keratinizing epithelium when cultured with irradiated mouse fibroblasts (Rheinwald and Green, 1975). Since then, various co-culture systems containing different types of cells have been designed and developed to study cell-cell communication, with direct or indirect cell-cell contact. However, these systems still rely on flat surfaces, restrict the extent of cell-cell interaction, and cannot replicate complex 3D structures of native tissues. In addition, the number of cell types that can be incorporated in such systems remains limited.

To better recapitulate the morphology, structure, function, and microenvironment of human tissues, 3D culture technologies have been developed, including spheroids and organoids. Spheroids are typically formed from immortalized cell lines or primary cells via self-aggregation and cell-cell adhesion (Kim et al., 2023). While spheroids provide uniform and reproducible structures, they lack the ability to differentiate and self-organize into organ-like architectures (Kim et al., 2023). In contrast, organoids are generated from tissue-specific stem cells or pluripotent stem cells (PSCs). Under appropriate biological stimuli, such as optimized growth factors and ECM components, these stem cells proliferate, differentiate, and self-assemble into 3D structures that mimic the architecture and function of the original tissue (Bar-Ephraim et al., 2020; Drost and Clevers, 2018; Hofer and Lutolf, 2021; Kim et al., 2020). With the advantages to closely recapitulate human tissue organization, organoids offer a robust and reproducible platform as animal models but keep human-specific features and can be applied in high-throughput applications. As a result, 3D biological models have become increasingly prominent in both basic and translational studies recently.

### Culture and expansion of primary HSCs

The function and frequency of HSCs was first demonstrated in 1958 by Till and McCulloh using a transplantation assay (Till and Mc, 1961). Shortly thereafter, Thomas performed the first successful human bone marrow transplantation between two identical twins (Armitage, 1994). To date, over 1 million patients have received bone marrow or HSC transplantation (Gratwohl et al., 2015; Niederwieser et al., 2022). However, the availability of suitable donors remains a major limitation due to the requirement of human leukocyte antigen matching, which often results in prolonged waiting time for patients. Therefore, efficient *in vitro* methods to generate, expand, and maintain functional HSCs are urgently needed for both clinical and research purposes (Ando et al., 2006; Antonchuk et al., 2002; Ema et al., 2000; Ghiaur et al., 2013; Rizo et al., 2008; Wilkinson et al., 2019).

Currently, a series of well-characterized surface markers have been developed to enable the high-purity isolation of HSCs from model organisms and human systems (Adolfsson et al., 2001; Civin et al., 1984; Dykstra et al., 2006; Kiel et al., 2005; Legras et al., 1997; Linnekin, 1999; Mayani and Lansdorp, 1994; Oguro et al., 2013; Spangrude et al., 1988; Vodyanik et al., 2006) for downstream applications such as culture, functional assay, and mechanistic studies (Ando et al., 2006; Antonchuk et al., 2002; Ema et al., 2000; Ghiaur et al., 2013; Rizo et al., 2008; Wilkinson et al., 2019). In conventional 2D culture systems, HSCs are maintained or expanded in the presence of key hematopoietic cytokines such as stem cell factor, thrombopoietin, and others (Ema et al., 2000; Wilkinson et al., 2019). Supportive niche cells, including MSCs, endothelial cells, or gene-modified cytokine-producing feeder cells have also been employed to sustain HSC cultures (Jing et al., 2010; Wilkinson et al., 2020; Zhang et al., 2023).

Recently, 3D culture systems using hydrogels or biomaterial scaffolds have been used to facilitate the expansion of HSCs *in vitro* (Bai et al., 2019; Liu et al., 2023; Zhou et al., 2020). While these systems represent a step forward, they typically lack the full complement of niche cells and organotypic morphological features. Therefore, despite their utility, the capacity and efficiency of both 2D and current 3D models remain unstable (Zhang et al., 2024a). The phenotypic markers and functional properties of cultured HSCs tend to change dynamically in these systems (Rubio-Lara et al., 2023; Zhang et al., 2024a), reducing the reliability and translational potential of these models. As a result, developing new technologies that faithfully model human hematopoietic tissues to produce and sustain functional HSCs *in vitro* remains an important mission in the field.

### Generation of HSPCs via gene modification

In parallel with advances in culture technologies, several methods using gene modification have been developed to reprogram non-hematopoietic cells into HSCs or other hematopoietic lineages (Table 1). For instance, overexpression of HoxB4, in combination with OP9 stromal co-culture or supplementation with extra cytokines, has been shown to confer HSC potential on embryonic stem cells (ESCs) and yolk sac-derived hematopoietic progenitors (Bowles et al., 2006; Kyba et al., 2002). Given that during embryonic development, hematopoietic stem/progenitor cells (HSPCs) originate from a special subset of arterial endothelial cells, raising the question of whether endothelial cells can be reprogrammed into HSCs *in vitro*.

**Table 1:** List and comparison of recent investigations about hematopoietic organoids

System	Studies	Cell/tissue resource?	Whether contain niche cells?	Whether contain EHT process?	Whether need gene editing?	Whether need extra cytokine supplement?	Whether can be maintained for long term?	Whether have special structures?	Whether embedded in hydrogel?	Whether constructed on a chip?	*In vivo* reconstitution test?
2D	Sandler et al. (2014)	Human umbilical vein endothelial cells	X	✓	✓	✓	✓	X	X	X	✓
	Szabo et al. (2010)	Human dermal fibroblasts	X	X	✓	✓	✓	X	X	X	✓
3D	Ng et al. (2016)	Human ESCs	✓	✓	X	✓	✓	✓	X	X	X
	Mao et al. (2016)	Human ESCs and human iPSCs	✓	✓	X	✓	✓	✓	X	X	X
	Motazedian et al. (2020)	Human ESCs and human iPSCs	✓	✓	X	✓	✓	✓	X	X	X
	Khan et al. (2023)	Human iPSCs	✓	✓	X	✓	✓	✓	✓	X	X
	Tamaoki et al. (2023)	Human iPSCs	✓	✓	X	✓	✓	✓	X	X	X
	Dardano et al. (2024)	Human iPSCs	✓	✓	X	✓	✓	✓	X	X	X
	Piau et al. (2023)	Human iPSCs	✓	✓	X	✓	✓	✓	X	X	✓
	Ng et al. (2025)	Human iPSCs	✓	✓	X	✓	✓	✓	X	X	✓
	Frenz-Wiessner et al. (2024)	Human iPSCs	✓	✓	X	✓	✓	✓	✓	X	✓ (Transient)
	Olijnik et al. (2024)	Human iPSCs	✓	✓	X	✓	✓	✓	✓	X	X
	Chou et al. (2020)	Donor-derived CD34^+^ cells, human umbilical vein ECs, human bone marrow-derived MSCs	✓	X	X	✓	✓	✓	✓	✓	X
	Georgescu et al. (2024)	Donor-derived CD34^+^ cells, human umbilical vein ECs, human bone marrow-derived MSCs	✓	X	X	✓	✓	✓	✓	✓	X

In 2014, Rafii and colleagues conducted the transcriptional comparison between lineage-negative (Lin^−^) CD34^+^ umbilical cord-derived HSPCs and human umbilical vein-derived endothelial cells (HUVECs). They identified 4 human HSPC-enriched transcription factors, FOSB, GFI1, RUNX1, and SPI1 (referred to as FGRS), as key regulators of hematopoietic identity. By using endothelial cells transduced with adenoviral E4ORF1 gene to build a vascular niche for the development of HSCs, they demonstrated that overexpression of FGRS in HUVECs or adult dermal microvascular endothelial cells can reprogram endothelial cells into hematopoietic cells with long-term MPP activity (rEC-hMPPs) (Lis et al., 2017; Sandler et al., 2014). These rEC-hMPPs were capable of engrafting and reconstituting hematopoiesis in irradiated immunocompromised mice (Lis et al., 2017; Sandler et al., 2014). Importantly, the roles of FGRS and vascular niche in driving the EHT are conserved across species. Overexpression of these factors, in combination with the supportive vascular niche, also transdifferentiates mouse adult endothelial cells into HSPCs with long-term engraftment capacity (Lis et al., 2017; Sandler et al., 2014).

Gene modification technologies have also been applied to generate engraftable HSPCs from PSC-derived HEs. Although several studies have successfully generated phenotypic HSPCs from human PSCs via intermediate HE stages, these cells often fail in *in vivo* reconstitution assays (Doulatov et al., 2013; Sugimura et al., 2017), suggesting that key regulators are missing during the conversion of HEs to HSPCs in PSC-based models. To address this limitation, Delay and colleagues combined PSC differentiation systems with gene modification approaches (Doulatov et al., 2013; Sugimura et al., 2017) and demonstrated that the expression of specific HSC-associated transcription factors is indispensable for generating bona fide HSPCs in PSC-derived models. By systematically screening 26 HSC-specific transcription factors, they identified a set of seven transcription factors (*ERG*, *HOXA5*, *HOXA9*, *HOXA10*, *LCOR*, *RUNX1*, and *SPI1*) that could sufficiently convert HEs into engraftable HSPCs capable of long-term multilineage reconstitution, including myeloid, B, and T cells, in primary and secondary mouse recipients (Sugimura et al., 2017).

Fibroblasts have also been used as a source for hematopoietic cell generation. For example, overexpression of OCT4, in the presence of hematopoietic supportive cytokines, successfully reprogrammed dermal fibroblasts into CD45^+^ hematopoietic cells (Szabo et al., 2010). In another study, overexpression of four transcription factors, Gata2, Gfi1b, cFos, and Etv6, reprogrammed fibroblasts into endothelial-like cells, which subsequently generated HSPC-like cells (Pereira et al., 2013).

Overexpression of c-MYC, BMI1, and BCL-XL enables human PSCs to establish immortalized megakaryocyte progenitor cell lines (imMKCLs) (Nakamura et al., 2014). With the aid of a turbulence-controllable bioreactor, imMKCLs can be cultured in a large-scale (8L) system, producing platelets on the order of 100 billion for potential clinical applications (Ito et al., 2018). In 2024, a clinical trial using autologous imMKCL-derived platelets was conducted in human patients, and no adverse symptoms or signs were observed following cell transfusion and during the subsequent 1-year follow-up period (Kayama and Eto, 2024).

While gene modification strategies offer a powerful and efficient approach to generate hematopoietic cells, they often involve genetically forced expression of transcription factors in a non-physiological manner. This limits their application in the investigation of natural developmental processes and raises safety concerns for clinical application due to potential off-target genetic effects. Nevertheless, the successful derivation of HSCs via an EHT-like process indicated that developmental pathways can be harnessed to inform the construction of HO models *in vitro*.

## Advances in HO studies

### Organoids recapitulate the architectures and function of organs

Organs are structural and functional units composed of spatially organized cell populations that collectively perform certain physiological functions (Drost and Clevers, 2018). The communication among different cell types within an organ guarantees the hemostasis maintenance and proper functions of the organ. In consequence, a comprehensive understanding of cellular behaviors and cell-cell interactions requires either intact primary tissues or *in vitro* culture systems that recapitulate the native tissue features.

Compared with model organisms, the accessibility to primary human tissue samples is quite limited, which significantly restricts the direct investigation of human biological process and hinders the examination of candidate drugs on patient-derived samples. Additionally, long-term *in vitro* maintenance of isolated human tissues is still a big challenge. To overcome these limitations, 3D organoid models have been developed in the past two decades and revolutionized the models of human organ development and diseases. The concept of an organoid was first demonstrated in 2009 by Clevers and his colleagues, who showed that a single Lgr5^+^ intestinal stem cell could spontaneously generate a whole crypt-villus-like multicellular structure in a laminin-rich Matrigel-based 3D culture system (Sato et al., 2009). Since then, multiple organoid culture methods have been established to model the development and pathology of various human tissues (Bar-Ephraim et al., 2020; Drost and Clevers, 2018; Hofer and Lutolf, 2021; Kim et al., 2020).

Organoid technologies have also been utilized in drug screening, particularly for cancer research. Traditional 2D culture of cancer cells isolated from murine or human patients has long been used in mechanistic studies and drug testing. However, these systems lack the morphological, biochemical, and biomechanical complexity of native tumors, thus making cultured cancer cells with different behavior and properties compared with their *in vivo* counterparts (Tebon et al., 2023), which often contributes to discrepancies between preclinical findings and clinical trial outcomes (Tebon et al., 2023).

Furthermore, 2D systems fail to capture the heterogeneity of *in vivo* cancer cells (Tebon et al., 2023), making it difficult to investigate or validate the responses to personalized medicines. To address these shortcomings, 3D patient-derived organoids (PDOs) have been developed as a promising platform to screen drugs for personalized anticancer therapy. Studies comparing drug responses across PDOs, xenografted models, and clinical trials found that PDOs efficiently represented the patient-specific responses observed *in vivo* (Vlachogiannis et al., 2018), suggesting that PDOs can serve as appropriate models for anti-cancer drug screening and precision medicine test.

Human PSCs, including human ESCs and human induced pluripotent stem cells (iPSCs), can serve as versatile cell resources for *de novo* organoid generation based on their accessibility and pluripotency. Prior to the appearance of iPSCs, ESCs were primarily used as the major cell source to establish protocols for the organoid construction (Corro et al., 2020; Eiraku et al., 2008; Hofer and Lutolf, 2021; Kim et al., 2020). The emergence of iPSCs (Takahashi and Yamanaka, 2006) has greatly accelerated the studies in developmental biology and regenerative medicine and made it theoretically possible to build organoids to recapitulate various organs in the whole human body.

Organoid generation from iPSCs shares some common intermediate stages (Hofer and Lutolf, 2021; Kim et al., 2020), including (1) iPSC seeding, (2) embryoid body (EB) formation, (3) trilineage germ layer differentiation, (4) lineage-specific cell differentiation (often involving vasculature induction), and (5) maintenance and maturation of the organoids. This modular framework has been successfully employed to construct organoids representing a variety of human tissues and organ systems (Hofer and Lutolf, 2021; Kim et al., 2020; McCauley and Wells, 2017), including digestive system (Crespo et al., 2017; Munera et al., 2019; Zhang et al., 2018a), nervous system (Lancaster and Knoblich, 2014; Lancaster et al., 2013), respiratory system (Dye et al., 2015; Hawkins et al., 2017; Jacob et al., 2017; Longmire et al., 2012; McCauley et al., 2017), urinary system (Morais et al., 2022; Taguchi et al., 2014; Takasato et al., 2014), hematopoietic system (Frenz-Wiessner et al., 2024; Khan et al., 2023; Motazedian et al., 2020; Ng et al., 2016, 2024; Olijnik et al., 2024), glands (Hayashi et al., 2022; Tanaka et al., 2022), and skin (Lee et al., 2020, 2022). These organoid systems have become indispensable tools for studying human development, modeling disease, and screening drugs and regenerative medicines.

### PSC-based HO generation

**Figure 1:**
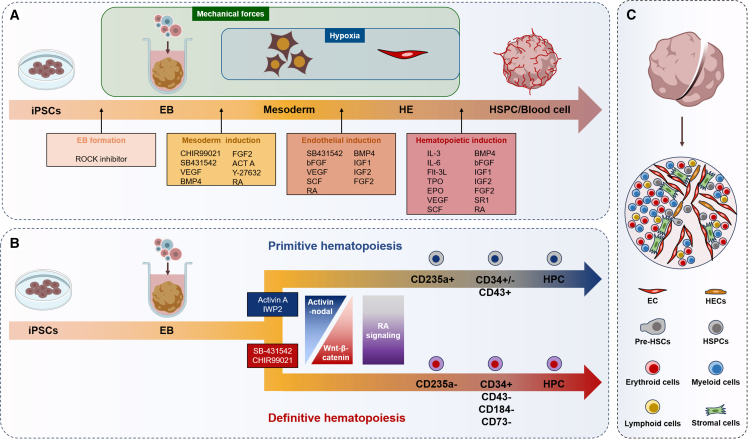
Current models for HO construction (A) Four-stage workflow to generate HOs from iPSCs: EB formation, mesodermal induction, endothelial induction, and hematopoietic induction. Cytokines that may be applied in each stage are listed. (B) Manipulation of Wnt-β-catenin signaling and Activin-nodal signaling determines the definitive and primitive hematopoietic specification from iPSCs. (C) Representative cell types and architectures existing in HOs. HOs mainly contain endothelial cells, stromal cells, hemogenic endothelium, pre-HSCs, HSPCs, and different types of differentiated hematopoietic cells.

Most studies of human HOs have been established using human PSCs, including ESCs and iPSCs (Table 1). The generation process of HOs is transgene free (Piau et al., 2023) and generally follows a stepwise differentiation process (Frenz-Wiessner et al., 2024; Khan et al., 2023; Ng et al., 2024; Olijnik et al., 2024), including (1) EB formation (Frenz-Wiessner et al., 2024; Khan et al., 2023; Ng et al., 2024; Olijnik et al., 2024), (2) mesodermal induction (Frenz-Wiessner et al., 2024; Khan et al., 2023; Ng et al., 2024; Olijnik et al., 2024), (3) vasculature and HE induction (Frenz-Wiessner et al., 2024; Khan et al., 2023; Ng et al., 2024; Olijnik et al., 2024), (4) hematopoietic induction (Frenz-Wiessner et al., 2024; Khan et al., 2023; Ng et al., 2024; Olijnik et al., 2024), and (5) maintenance phase (Frenz-Wiessner et al., 2024; Khan et al., 2023; Ng et al., 2024; Olijnik et al., 2024) (Figure 1A). Depending on the protocol applied during the differentiation, HOs and HO-derived HSCs can give rise to multiple types of hematopoietic progenitor cells and differentiated cells (Frenz-Wiessner et al., 2024; Khan et al., 2023; Mao et al., 2016; Ng et al., 2024; Olijnik et al., 2024). Although many HO generation protocols follow a similar framework, and are largely similar among different reports, several different aspects still exist, such as important differences in the types and concentrations of specific factors (e.g., vascular endothelial growth factor [VEGF] and Activin A) and the employment of hydrogels, hypoxia conditions, and extra supportive niche cells (Dardano et al., 2024; Frenz-Wiessner et al., 2024; Khan et al., 2023; Ng et al., 2024; Olijnik et al., 2024; Rezvani et al., 2024).

During hematopoietic development, two sequential waves are recognized: primitive hematopoiesis, which mainly produces erythroid progenitors and mature hematopoietic cells, and definitive hematopoiesis, which gives rise to bona fide long-term functional HSCs (Ivanovs et al., 2017). In HO systems, the balance between these two programs can be modulated by signaling cues, which determine the fate and features of emerging hematopoietic cells (Kennedy et al., 2012; Sturgeon et al., 2014). A well-characterized example involves the Activin/Nodal signal and Wnt–β-catenin signaling pathway. Activation of Activin/Nodal signal and inhibition of Wnt-β-catenin pathway promote primitive hematopoiesis. In contrast, low Activin/Nodal signal combined with Wnt-β-catenin pathway activation favors definitive hematopoietic specification (Kennedy et al., 2012; Sturgeon et al., 2014) (Figure 1B). Thus, precise modulation of these pathways is crucial for optimizing HSC outputs in HOs.

VEGF is a critical regulator of endothelial and vascular development in organoids. As HSCs originate from HEs (Frenz-Wiessner et al., 2024), which are closely related to arterial endothelial cells, VEGF is essential for early vascular and HE formation in HOs. However, sustained VEGF signaling was found to inhibit the hematopoietic transition of HEs by suppressing key hematopoietic transcription factors, such as *Runx1*, *Fli1*, and *Gata* (Edginton-White et al., 2023). As expected, several studies reported that reducing the concentrations of VEGF in hematopoietic induction and maintenance phases significantly facilitated the generation of HSCs in HOs (Frenz-Wiessner et al., 2024; Khan et al., 2023; Liang et al., 2025; Ng et al., 2024; Olijnik et al., 2024). In one study, complete removal of VEGF during hematopoietic induction and maintenance phases significantly improved the reconstitution capacity of HO-derived HSCs in immunodeficient mice (Ng et al., 2024).

The ECM is the non-cellular macromolecule network that provides critical structural and biochemical support for tissue development and maintenance. To mimic the ECM microenvironment, some groups have embedded organoids in collagen-containing hydrogels (Frenz-Wiessner et al., 2024; Khan et al., 2023; Olijnik et al., 2024). After mesodermal induction, organoids can be embedded in hydrogel for vascular sprouting and hematopoietic induction (Frenz-Wiessner et al., 2024; Khan et al., 2023; Olijnik et al., 2024). Although detailed mechanistic studies are still lacking, hydrogels likely function by improving 3D structures and promoting ECM-integrin signaling and potentially promote HSPC emergence in HOs.

Retinoic acid (RA) signaling plays important roles in many developmental lineages, functioning as a morphogen to regulate cell fate specification, proliferation, and differentiation (Cunningham and Duester, 2015). RA signaling has been shown to inhibit primitive hematopoiesis and facilitate definitive hematopoiesis (Chanda et al., 2013; de Jong et al., 2010), and the manipulation of RA signaling markedly influences hematopoietic differentiation form PSCs (Chanda et al., 2013; Ronn et al., 2015). Notably, activation of RA signaling during the construction of AGM-like organoids enhances the reconstitution potential of iPSC-derived hematopoietic cells (Ng et al., 2024).

Mechanical cues represent fundamental regulators of hematopoietic development (North et al., 2009; Vining and Mooney, 2017; Wang et al., 2011); blood flow-induced forces have been shown to be essential for hematopoiesis in the zebrafish AGM region (North et al., 2009; Wang et al., 2011). A recent study demonstrated that transient activation of the mechanosensitive iron channel Piezo1 via Yoda1 facilitated *de novo* generation of long-term repopulating HSCs from human PSCs (Scapin et al., 2025). Likewise, oxygen tension influences hematopoietic development; hypoxic conditions benefit the stem cell proliferation, survival, and differentiation. In some studies, EBs were treated under hypoxia during mesodermal induction phase, followed by normoxia condition in the following steps (Kennedy et al., 2012; Olijnik et al., 2024; Sturgeon et al., 2014). Additionally, niche cells play indispensable roles in hematopoietic development and maintenance. Several studies applied co-culture of HOs with different types of supportive niche cells and significantly promoted hematopoiesis (Zhang et al., 2023).

The air-liquid interface (ALI) technique, in which cells or tissues are cultured with their basal surface in contact with liquid culture medium and their apical surface exposed to air (Han and Zuniga-Pflucker, 2021), provides another promising approach for hematopoietic modeling. Beyond its common use for airway epithelial cell culture, the ALI technique has been used to maintain certain hematopoietic tissues *in vitro*, such as AGM region and thymus organ (Han and Zuniga-Pflucker, 2021; Lv et al., 2017). The system allows for high oxygen exposure, a condition crucial for thymocyte differentiation in cultured thymus organs (Han and Zuniga-Pflucker, 2021). Based on this strategy, functional thymic organoids derived from human PSCs have been successfully established, producing both thymic epithelial cells and T cells (Ramos et al., 2023). Moreover, ALI-based culture of AGM tissues can preserve tissue architecture and continuous HSC generation for 2–4 days *in vitro* (Fitch et al., 2012; Lv et al., 2017). However, the effect of ALI system on AGM-like organoid has not been evaluated. Given the beneficial effects of ALI culture on maintaining AGM architecture and promoting thymocyte maturation, integrating ALI systems into HO differentiation protocols may promote the EHT efficiency and improve the multipotent capacity of newly generated HSPCs.

With diverse combinations of signaling cues, culture matrices, and niche components, researchers have successfully developed HOs that mimic specific hematopoietic tissues, including the AGM region (Dardano et al., 2024; Ng et al., 2016), yolk sac (Tamaoki et al., 2023; Zambidis et al., 2005), fetal liver (Rezvani et al., 2024), bone marrow (Frenz-Wiessner et al., 2024; Khan et al., 2023; Olijnik et al., 2024), and thymus (Fan et al., 2015; Ramos et al., 2023; Seet et al., 2017). These organoids shared key features (Table 1), such as undergoing EHT process for HSC generation and partially reflected characteristics of native hematopoietic tissues. These systems have advanced our understanding of hematopoietic development, maintenance, and diseases. In the future, by studying across different HO platforms and systematically evaluating the roles of signaling inputs, matrix components, and cellular composition, the fidelity and utility of HOs for both basic research and clinical translation can be further improved.

### Hematopoietic niche cells in 3D models

Besides the soluble cytokines and biofunctional factors, the *in vivo* maintenance of HSCs requires special support from the surrounding niche cells. *In vitro* culture models have been used to explore the properties and regulatory mechanisms of these hematopoietic supportive niche cells, particularly MSCs and endothelial cells. As discussed above, incorporation of niche cells into the HSC culture system has been shown to improve HSC expansion and maintenance (Zhang et al., 2023).

MSCs can be isolated from various tissues, including bone marrow, adipose tissue, umbilical cord, and gingiva and cultured *in vitro* (Zhou and Shi, 2023). However, in conventional 2D culture systems, bone marrow-derived MSCs (BMSCs) lost their expression of critical hematopoietic supportive cytokines such as *Scf* and *Cxcl12*, contributing to their loss of capacity to maintain HSCs (Zhang et al., 2023). The decrease of *Scf* and *Cxcl12* expression is largely attributed to the high stiffness of 2D culture, as culturing BMSCs in soft 3D matrix restored the expression of *Scf* and *Cxcl12* in BMSCs and rescued their capacity to support HSC maintenance and lymphopoiesis (Zhang et al., 2023).

Additionally, MSCs in 3D cultures exhibit improved paracrine signaling, enhanced self-renewal capacity, and better multipotency, contributing to superior therapeutic efficacy in applications such as immunomodulation and wound healing (Bartosh et al., 2010; Potapova et al., 2007; Zhang et al., 2015b; Zhou et al., 2017b). These advantages highlight the importance of biomechanical and spatial cues in preserving the functional phenotype of MSCs *in vitro*.

Culture of endothelial cells also benefits significantly from 3D systems. Endothelial cells are critical regulators of the vascular niche, which produces a wide range of factors essential for the proper maintenance of perivascular cell types (Afshar et al., 2023; Comazzetto et al., 2019; Ding et al., 2012), including HSCs (Comazzetto et al., 2019; Ding et al., 2012). In 2D culture systems, endothelial cells form monolayers with altered transcriptome and, therefore, fail to recapitulate the *in vivo* vascular structure (Afshar et al., 2023). In contrast, organoid culture systems enable vascular assembly and vascular lumen formation, in the presence or absence of hydrogels or particular supporting cells (Nguyen et al., 2013; Shamir and Ewald, 2014; Zheng et al., 2012). Improved functional vasculature has been found to promote the formation and maintenance of different organoids (Naderi-Meshkin et al., 2023; Worsdorfer et al., 2019).

### HO-on-a-chip

In addition to PSC-derived organoids, HOs can also be constructed using primary tissues or cells on microfluidic platforms, commonly referred to as organ-on-a-chip systems.

In 2014, Ingber and his colleagues pioneered a bone marrow-on-a-chip model by seeding murine bone marrow cells onto microengineered chips. Such models successfully maintained the properties and function of HSPCs for at least 1 week (Torisawa et al., 2014) and could be used to screen potential radioprotective agents (Torisawa et al., 2014, 2016).

Building on this success, the same team, by seeding human-derived cells, including CD34^+^ cells, BMSCs, and human umbilical vein endothelial cells on a chip, built vascularized human bone marrow-on-a-chip (Chou et al., 2020). Not only used for toxicity examination, similar models can also be used to recapitulate hematopoietic abnormalities in patients with genetic mutations (Chou et al., 2020) or be applied to test the toxicity of chemotherapy drugs and help optimize the doses of the drugs in clinical trials (Georgescu et al., 2024). Furthermore, a recent study employed similar models to investigate the mobilization of bone marrow myeloid cells upon stress or tissue injury. After seeding on a chip with endothelial cells, MSCs, and fibroblasts, human CD34^+^ HSPCs exerted multilineage hematopoiesis, including the generation of mature myeloid cells. When the systems were treated with ionizing radiation, or linked to lipopolysaccharide-treated lung organoids, the mature myeloid cells became motile and migrated across the endothelial barrier (Georgescu et al., 2024).

Compared to the PSC-based HOs, the major obstacle of primary cell-based human HOs is the difficulty in obtaining fresh samples. As HO-on-a-chip mimics certain features of hematopoietic tissues and the construction method is different from that of PSC-derived HOs, a more detailed comparison between PSC-based HOs and primary cell-based HO-on-a-chip is warranted, which will provide more useful information on the construction principles of hematopoietic tissues and will help optimize the HO systems to become more authentic.

## The applications of HOs

### Studying human hematopoiesis

**Figure 2:**
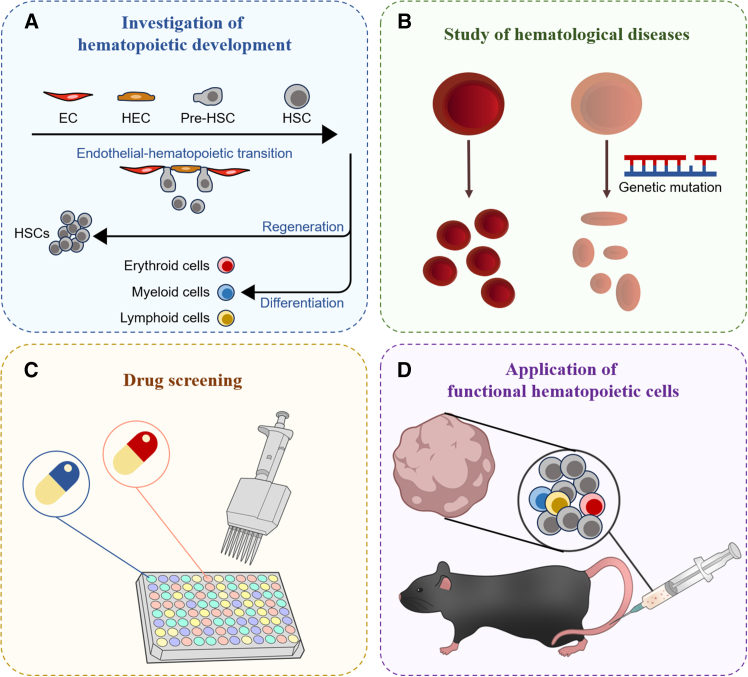
The applications of HOs HOs can be employed in (A) investigation of hematopoietic development, (B) studies of hematopoietic diseases, (C) drug screening for hematopoietic disorders, and (D) generation of functional hematopoietic cells (including HSPCs and differentiated hematopoietic cells) for basic research and clinical trials.

Human HOs provide efficient and physiological models for the investigation of human hematopoiesis (Frenz-Wiessner et al., 2024; Khan et al., 2023; Ng et al., 2016; Olijnik et al., 2024) (Figure 2A). In iPSC-derived HOs, HEs emerge under defined induction protocols and subsequently undergo EHT to generate HSPCs (Frenz-Wiessner et al., 2024). By manipulating cytokine combinations during differentiation, scientists have generated HSPCs with either primitive properties or definitive hematopoietic fate (Kennedy et al., 2012; Ng et al., 2016; Sturgeon et al., 2014; Tamaoki et al., 2023; Zambidis et al., 2005). Single-cell RNA sequencing (scRNA-seq) has been used to compare HO-derived HSCs with their *in vivo* counterparts (Ng et al., 2024; Tamaoki et al., 2023; Zambidis et al., 2005). Although there are still some transcriptional differences remaining, the key gene expression profiles during EHT are largely conserved (Ng et al., 2024; Tamaoki et al., 2023; Zambidis et al., 2005). Functionally, HO-derived HSPCs are capable of generating myeloid, lymphoid, and erythroid cells in both *in vitro* culture and xenografted animal models (Ng et al., 2016; Tamaoki et al., 2023).

Beyond early emergence, HOs also provide opportunities to study the maturation of HSCs during development. Fetal liver is a transient but critical niche where HSCs undergo maturation, expansion, and differentiation (Gao et al., 2022; Khan et al., 2016; Xue et al., 2017). In a recent preprint paper, investigators co-induced endodermal and mesodermal differentiation from human PSCs to simultaneously generate yolk sac-like HEs and hepatocytes, thus constructing fetal liver-like organoids (Rezvani et al., 2024). These fetal liver-like organoids are capable of producing hematopoietic progenitor cells, which can subsequently give rise to myeloid, lymphoid, and erythroid cells, and the hematopoiesis maintenance of these organoids is self-sufficient (Rezvani et al., 2024).

Similarly, bone marrow-like organoids have been developed (Frenz-Wiessner et al., 2024; Khan et al., 2023; Olijnik et al., 2024) to model and study adult hematopoiesis. These organoids contain endothelial cells, stromal cells, HSPCs, and differentiated hematopoietic cells (Frenz-Wiessner et al., 2024; Khan et al., 2023; Olijnik et al., 2024) and can be maintained long-term *in vitro* (Frenz-Wiessner et al., 2024; Khan et al., 2023; Olijnik et al., 2024). As the bone marrow-like organoids resemble the architecture and function of native bone marrow (Frenz-Wiessner et al., 2024), these models are well suited to study the homeostasis, diseases, and regeneration in adult bone marrow.

### Modeling hematopoietic diseases

In addition to modeling physiological hematopoiesis, HOs can be applied for the study of hematopoietic diseases (Figure 2B), including fibrosis, hematopoietic malignancy, and bone marrow transplantation.

Bone marrow fibrosis often co-occurs with hematopoietic malignancy, and transforming growth factor β (TGFβ) signaling was shown to induce bone marrow fibrosis-like phenotypes in HOs (Khan et al., 2023; Olijnik et al., 2024). Engraftment of cells from myelofibrosis patients, but not healthy donors, elevated TGFβ levels and induced fibrosis in organoids, which could be inhibited by TGFβ inhibitors (Khan et al., 2023). These studies demonstrated the potential of HOs as models for investigating disease pathophysiology and evaluating antifibrotic therapies (Figure 2C).

Genetically engineered HOs can recapitulate patient-specific disease phenotypes. For instance, the deficiency of vacuolar protein sorting 45 homolog (*VPS45*) has been found to cause neutropenia and myelofibrosis in children. To model *VPS45* mutation-induced hematopoietic diseases, investigators generated *VPS45*-mutated iPSCs (Frenz-Wiessner et al., 2024) and found that *VPS45*-deficient iPSC-derived HOs reproduced the disease features observed in patients (Frenz-Wiessner et al., 2024), validating the potential use of genetically modified HOs in studying gene mutation-associated hematopoietic disorders.

HOs can also be used as platforms to assess HSPC engraftment and investigate hematopoietic malignancies. Various studies have demonstrated the successful engraftment of patient- or donor-derived HSPCs, malignant hematopoietic cells, and hematopoietic cell lines into pre-established HOs (Giger et al., 2022; Olijnik et al., 2024; Ren et al., 2025). In one study, HOs supported the maintenance and growth of CD34^+^ leukemic blasts (Giger et al., 2022). Furthermore, the migration of leukemic blasts into HOs is more rapid than that of normal CD34^+^ HSPCs (Giger et al., 2022). In another study, by separately engrafting HSPCs derived from healthy people or myelodysplastic syndrome patients into human HOs, investigators found that although HOs facilitated the hematopoiesis of both kinds of HSPCs, only MDS HSPCs could influence the HO status and recapitulate MDS pathology within them (Ren et al., 2025).

These investigations collectively demonstrated the potential of HOs to function as disease models for drug screening, mechanistic studies, and personalized therapeutic evaluation (Figure 2C).

### Generating functional hematopoietic cells

Recent studies demonstrated that HOs can generate multiple kinds of functional and transplantable hematopoietic cells, including HSCs, T cells, and macrophages (Frenz-Wiessner et al., 2024; Ng et al., 2024; Ramos et al., 2023), making the system with greatly promising to fulfill the clinical requirement of various hematopoietic cells (Figure 2D).

Although not yet tested clinically in humans, HSCs generated in PSC-based AGM-like organoids robustly reconstituted hematopoiesis in both bloodless zebrafish embryos and immunodeficient mice (Chang et al., 2022). Using a design-of-experiment approach, a transgene-free and stroma-free method was established to improve the efficiency of PSC-derived HSC production by systematically comparing 16 different protocols (Piau et al., 2023). In this system, PSC-derived EB cells (4 × 10^5^ per mouse) were capable of robust, long-term, and multipotent hematopoietic reconstitution in immunocompromised mice (Piau et al., 2023). The multilineage reconstruction was observed in 59 of 60 primary recipient mice and all 40 secondary recipients (Piau et al., 2023).

Mesodermal patterning of ESCs with CHIR99021 and SB431542 generated CD34^+^ hematopoietic cells expressing HOXA genes, closely resembling human AGM-derived hematopoietic cells (Ng et al., 2016). Recently, scientists successfully generated long-term engrafting multilineage HSCs from PSCs by conducting HOXA patterning and optimizing factor combination and concentration in a chemically defined differentiation strategy (Ng et al., 2024). This study demonstrated that the addition of retinyl acetate, an RA precursor, into hematopoietic differentiation medium enhanced the long-term, multilineage reconstitution capacity of HO-derived HSCs (Ng et al., 2024). Furthermore, VEGF withdrawal after the vascular induction phase promoted the efficiency of EHT and improved the engraftment potential of newly generated HSCs (Ng et al., 2024). With these refinements, HO-derived HSCs achieved 25%–50% of multilineage hematopoietic reconstitution in immunodeficient mice. However, the required cell input remains high, about 0.5–2 million (500,000–2,000,000) CD34^+^ cells for each mouse. As a comparison, human umbilical cord-derived CD34^+^ cells can achieve more than 90% reconstitution efficiency with as few as 6,000 cells per mouse (Ng et al., 2024). Consistently, when only 1 × 10^5^ HO-derived CD34^+^ cells were transplanted into immunodeficient mice, the multilineage reconstitution was observed in only 30% of recipients. Thus, sufficient cell number and functional maturation are likely critical for successful engraftment of HO-derived HSPCs (Frenz-Wiessner et al., 2024).

Mechanical signaling has emerged as another key determinant of HSC generation (North et al., 2009; Wang et al., 2011). Consistent with findings in the AGM region, circumferential stretch promoted the generation of long-term engrafting multilineage HSCs from human PSC-based HOs (Scapin et al., 2025). Hematopoietic cells generated without mechanical stimulation failed to perform hematopoietic reconstitution, whereas transplantation of 100 000 cells derived under circumferential stretch or Piezo1 activation successfully reconstructed multilineage hematopoiesis in both primary and secondary recipient mice (Scapin et al., 2025). In another preprint study, human PSC-derived fetal liver-like organoids were transplanted into the kidney capsule of immunodeficient mice and generated multiple types of cells, including CD34^+^CD45^+^ cells (Rezvani et al., 2024).

Based on the above-mentioned studies, HO-derived HSCs have been proved to be capable of hematopoietic reconstruction; however, the reconstitution efficiency of organoid-derived HSCs still needs to be improved for future clinical utilization. Systematic multi-omics and functional comparison among HSCs generated by different protocols will be helpful to identify conditions for bona fide HSC generation. Moreover, direct comparison between *in vivo* tissues and HOs may reveal additional microenvironmental signals critical for the generation and expansion of functional HSCs *in vitro*.

Beyond HSCs, HOs have also been used to generate functional immune cells, such like T cells and macrophages (Iriguchi et al., 2021; Montel-Hagen et al., 2019; Ramos et al., 2023; Seet et al., 2017; Shen et al., 2024). By treating the organoids or HO-derived HSPCs with specific cytokines, lineage-specific differentiation can be guided. Such HO-derived immune cells have exhibited functional activity and may serve in immunotherapy for the treatment of immune disorders and cancers in the future (Iriguchi et al., 2021; Montel-Hagen et al., 2019; Shen et al., 2024).

## Challenges and future directions

### Functional HSPCs from HOs remain suboptimal

While HSPCs derived from organoids have been demonstrated to be capable of multi-lineage hematopoietic reconstruction in xenografted models, their reconstitution efficacy remains markedly lower than that of cord blood-derived HSPCs (Ng et al., 2024). This low reconstitution efficiency may be attributed to the incomplete maturation and cellular heterogeneity of HO-derived HSPCs, which may reflect the suboptimal microenvironmental cues in organoids.

**Figure 3:**
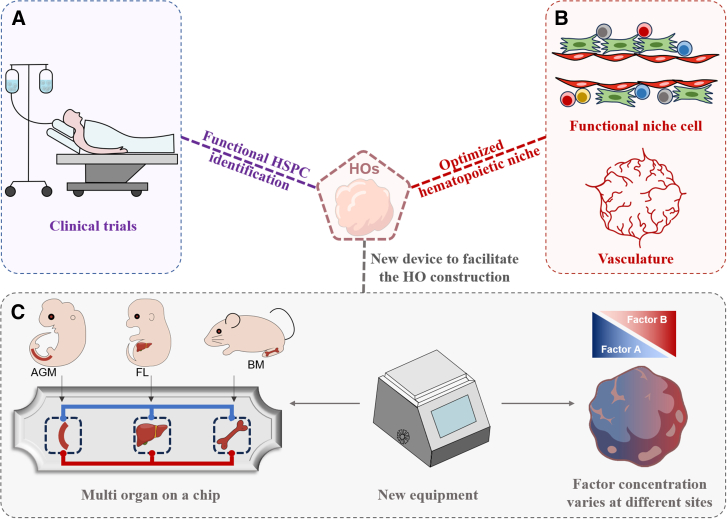
Potential approaches to improve HO models (A) After establishing new protocols to identify and isolate functional hematopoietic cells from HOs, these cells are expected to be employed in clinical trials. (B) Improve the function of niche cells and optimize the vasculature within HOs. (C) Develop new equipment (with better microfluidics system), to precisely regulate the types, concentrations, and spatial distribution of factors used during differentiation, and construct “*in vitro* hematopoietic systems” through connecting different types of HOs.

Extra culture in the presence of specific hematopoietic factors may improve the maturation of HO-derived HSPCs, reflected by the appearance of hematopoietic progenitor cells and mature cells (Olijnik et al., 2024). Manipulation of EHT regulators, such as VEGF and RA, has been shown to influence the emergence and reconstitution capacity of HO-derived HSPCs. Therefore, a precise control of types and concentration of factors during the differentiation should benefit the maturation of HSPCs (Ng et al., 2024). Nevertheless, the optimal combination and temporal control of factors for the proper maturation of HO-derived HSPCs are still largely unknown. To solve these problems, spatial transcriptomics and high-resolution scRNA-seq should be performed to (1) compare and track the generation and maturation of HSPCs within organoids to enhance the reproducibility of existing protocols, (2) identify unique markers distinguishing bona fide functional HSPCs from non-functional CD34^+^ populations, and (3) determine whether hematopoietic development follows spatial and temporal patterns as it occurs *in vivo*. Comparative multi-omics analyses between HO-derived and tissue-derived HSPCs will be essential to reveal the mechanisms underlying lower engraftment efficiency and to inform strategies to enhance functional HSPC outputs (Figure 3A).

### Niche optimization in hematopoietic organoids

Currently, the properties, function, and spatial distribution of each kind of niche cell in HOs have not been fully studied and the comparative studies of niche cells from HO and native tissues are still lacking. Compared to native hematopoietic tissues, HOs lack the full complement and appropriate proportions of niche cell types such as endothelial cells, stromal cells, osteoblastic cells, and neuronal cells (Frenz-Wiessner et al., 2024; Khan et al., 2023; Olijnik et al., 2024). Considering the important supportive and regulatory functions of niche cells in hematopoiesis through the whole life (Comazzetto et al., 2021; Ding et al., 2012; Morrison and Scadden, 2014), their incomplete or immature representation in HOs may limit authentic HSC generation and maintenance. For instance, optimized fetal liver-like organoids should involve functional fetal hepatocytes (Rezvani et al., 2024) and the maturation of fetal hepatocytes may need to cooperate with the maturation of HSPCs. For bone marrow-like organoids, functional MSCs, endothelial cells, osteoblasts, chondrocytes, and neurons are all needed to provide essential supportive signals and ECM for HSC maintenance (Figure 3). As current methods for HO construction are based on very limited kinds of cytokines and factors, the types and function of niche cells generated in HOs may be largely different from those *in vivo*. To address this, niche cells can be generated from PSCs separately and added at specific differentiation phases to enrich organoid complexity. Furthermore, based on the hematopoietic organ of interest, the types and the ratio of niche cells should be tailored to assemble desirable hematopoietic microenvironments (Figure 3B). Recently emerged multilineage co-differentiation technology enables the simultaneous generation of mesodermal and endodermal cells within the same organoid (Miao et al., 2025); the similar multilineage co-development system may also be applied to provide more types of niche cells and enable the generation of fully functional HOs.

Endothelial cells, in particular, are central to HSC development, as they not only support hematopoiesis but also give rise to HSCs via EHT per se (Frenz-Wiessner et al., 2024; Khan et al., 2023; Ng et al., 2016; Olijnik et al., 2024). Hence, improved vasculature could largely improve HO performance. Several strategies can be applied to promote the vasculature in HOs, including modulation of vascular signaling pathways (e.g., Notch and VEGF) and incorporation of hydrogels or 3D printing scaffolds (Figure 3B).

To study the genetic and signaling pathways involved in the construction of human blood vessel organoids (hBVOs), recent studies analyzed hBVOs with paired scRNA-seq and single cell assay for transposase-accessible chromatin using sequencing (scATAC-seq) (Nikolova et al., 2025). The researchers found that arterial-like endothelial cells appeared much earlier (at around ray 6) than venous endothelial cells (at around day 21) (Nikolova et al., 2025), which is consistent with the reported time point for the happening of EHT progress in HOs (at around day 7) (Ng et al., 2024). Furthermore, they found that endothelial cells exhibited organ-specific transcriptional features, and their hBVO-derived endothelial cells were more similar to pancreas-derived endothelial cells than those from other organs (including muscle, intestine, stomach, and brain) (Nikolova et al., 2025). Notably, they identified an instructive role of MDS1 and EVI1 complex locus protein (MECOM) in regulating the development of both vasculature and hematopoiesis. This information indicated that vasculature in HOs could be tailored toward tissue-specific endothelial phenotypes by fine-tuning transcriptional regulators and the generation of hematopoietic tissue-specific endothelial cells should significantly facilitate HO construction accordingly.

Based on their morphology, blood vessels can be distinguished as arterioles, veins, and sinusoids. In different hematopoietic organs, perivascular niches based on different kinds of vessels have been identified to support HSPCs and hematopoiesis in different manners (Agrawal et al., 2024; Comazzetto et al., 2021; Khan et al., 2016; Morrison and Scadden, 2014; Morrison et al., 1995). The application of 3D scaffolds may help build different types of blood vessels and provide a more physical perivascular niche for HOs. While endothelial cells can be monitored in current organoid systems, the morphologies of blood vessels in organoids are not identical. To overcome the limitation, arterial endothelial cells and sinusoidal endothelial cells may need to be added into 3D scaffolds in an optimized ratio, to build different perivascular niches to facilitate the construction of HOs.

Hydrogel was first highlighted and used for mammary gland formation in 1987, and many organoids are cultured in hydrogel-based 3D systems now (Barcellos-Hoff et al., 1989; Bissell and Barcellos-Hoff, 1987; Li et al., 1987; Petersen et al., 1992). Different collagens are contained in hydrogel, which are used to mimic the *in vivo* extracellular microenvironment and to promote the survival, proliferation, and maintenance of cultured cells. The application of collagen-enriched hydrogel in organoid systems has efficiently promoted the vascular vessel sprouting of organoids (Frenz-Wiessner et al., 2024).

### Precise HO construction and their integration need the support from new instruments

Compared to traditional 2D culture systems, HOs more effectively emulate the complexity of native hematopoietic tissues. However, further refinement of spatial and temporal control during differentiation is required to make the HOs truly organ-like. To achieve this, the development and application of advanced instruments capable of creating more physiological microenvironment for HO construction are urgently needed.

Currently, HO construction mainly depends on a limited set of cytokines and morphogens to induce stepwise differentiation of PSC into hematopoietic cells (Dardano et al., 2024; Frenz-Wiessner et al., 2024; Khan et al., 2023; Ng et al., 2016, 2024; Olijnik et al., 2024). In contrast, *in vivo* native hematopoietic tissues are orchestrated by a far more complex and dynamic biochemical environment, with finely tuned gradients of soluble factors, ECM components, biomechanical cues, and vascular perfusion. These physical and chemical signals jointly regulate cell polarity, fate commitment, and functional specialization (Zhang et al., 2024b). Mimicking these aspects through engineered systems could substantially improve the fidelity, maturity, and functionality of HOs (Figure 3).

Emerging evidence supports the idea that co-culturing organoids with distinct tissue types enhances their development and sustains their functionality by mimicking organ-organ communication *in vivo* (Dardano et al., 2024; Rezvani et al., 2024). This is particularly relevant to the hematopoietic system, which involves multiple interrelated organs and tissues, including AGM, fetal liver, bone marrow, spleen, thymus, and lymph node, all of which work collaboratively to maintain hematopoiesis (Calvanese et al., 2022; Doulatov et al., 2012; Ivanovs et al., 2017; Kondo et al., 2003; Morrison and Scadden, 2014; Orkin and Zon, 2008). New microfluidics systems may help build *in vitro* “hematopoietic systems” to reflect different hematopoietic continua. As an example, AGM-like organoids, fetal liver-like organoids, and bone marrow-like organoids can be sequentially connected to recapitulate early HSC emergence, expansion, differentiation, and migration during development (Figure 3). Similarly, bone marrow-, thymus-, and spleen-like organoids can be integrated to mimic hematopoietic homeostasis, immune maturation, and hematopoietic regeneration. Considering the differences between HOs and *in vivo* tissues, the order and timing of HO integration should be carefully evaluated to ensure that the resulting system accurately represents *in vivo* hematopoietic networks.

Organ-on-a-chip technologies have already shown great promise in simulating organ physiology *in vitro.* Organ-on-a-chip has several advantages compared to conventional organoid culture systems including (1) autonomous medium change and waste removal, (2) adjustable mechanical stimulation and better structures for vasculature, and (3) allowing structured integration of multiple organoids on the chip. Although still in its infancy, application of organ-on-a-chip technologies to HO modeling could promote the development of the field. For example, organ-on-a-chip can provide the heart beating-like mechanical stimulation, which mimics the mechanical properties of dorsal aorta within the AGM region, and may better support the construction of AGM-like organoids. In potential integrated systems, different types of HOs can be established and linked on one chip, as described above, to recapitulate the continuous, multistage nature of human hematopoiesis across development or adult contexts. Such platforms would not only enhance the physiological relevance of HO models but also open new avenues for studying inter-organ signaling, modeling systemic hematological diseases, and screening drugs in personalized medicine settings.

## Future directions

Since the advent of 3D organoid models, numerous studies have successfully constructed HOs starting from human PSCs or primary cells. By incorporating specific cytokines, bioactive factors, and functional niche components in the differentiation system, researchers have developed different HOs with specific features, including AGM-like, yolk sac-like, fetal liver-like, and bone marrow-like organoids. These HO models provide tractable models to investigate human hematopoiesis development and maintenance. Furthermore, HOs can also be used as efficient models for pathological studies and drug screening, through approaches like genetic engineering and engraftment of patient-derived samples.

Despite these advances, major challenges remain. In particular, the generation of bona fide functional HSCs with long-term multilineage reconstitution capacity is still limited. Many critical questions should be addressed to advance the field. (1) What mechanisms underlie the reconstitution capacity differences between organoid-derived and native tissue-derived HSCs? (2) How can we ensure the reproducibility of HO differentiation from PSCs and primary progenitor cells? (3) How can we efficiently build stable hematopoietic disease-specific organoids for investigation of hematopoietic disorders? (4) How can we construct a comprehensive, *in vitro* “hematopoietic system” that mimics the full spectrum of human hematopoiesis? (5) How can HO models be standardized for drug screening, precision medicine, and clinical applications?

To overcome these challenges, future studies should incorporate cutting-edge technologies, such as (1) single-cell multi-omics and spatial transcriptomics, which can be employed to dissect cellular heterogeneity and spatial organization within individual HO, as well as to compare HOs generated by different construction protocols, thereby improving the reproducibility and standardization of HO models; (2) 3D bioprinting and engineered scaffolds to create more anatomically structured and physiologically relevant niches; and (3) organoid co-culture platforms and organ-on-a-chip systems to provide dynamic, perfused, and mechanically active environments and mimic inter-organ communication.

The integration of these technologies with current HO methodologies will be essential to improving their functionality, fidelity, and translational potential. Ultimately, fully optimized HOs may serve not only as powerful tools for basic and translational hematology research but also as next-generation platforms for personalized therapy, regenerative medicine, and hematopoietic disease modeling.

## Acknowledgments

The authors thank Dr. Dongyuan Ma and Dr. Yifan Zhang for their invaluable thinking on the draft of this manuscript. This work was supported by grants from the 10.13039/501100001809National Natural Science Foundation of China (32530034, 92468201, and 32030032), the 10.13039/501100012166National Key R&D Program of China (2023YFA1800100), and the Initiative Scientific Research Program, 10.13039/501100011186Institute of Zoology, Chinese Academy of Sciences (2023IOZ0305 and 2023IOZ0102).

## Author contributions

F.L., L.D. conceived the manuscript idea. F.L., L.D., and Y.H. wrote the manuscript. All authors approved the final version of the manuscript.

## Declaration of interests

F.L. is a member of the editorial board of Stem Cell Reports.
